# Biomarkers and Hematological Indices in the Diagnosis of Iron Deficiency in Children with Inflammatory Bowel Disease

**DOI:** 10.3390/nu12051358

**Published:** 2020-05-09

**Authors:** Paulina Krawiec, Elżbieta Pac-Kożuchowska

**Affiliations:** Department of Pediatrics and Gastroenterology, Medical University of Lublin, Al. Racławickie 1, 20-059 Lublin, Poland; elzbietapackozuchowska@umlub.pl

**Keywords:** Crohn’s disease, ulcerative colitis, iron depletion, anemia

## Abstract

Inflammation may affect many routinely available parameters of iron homeostasis. Thus, the recognition of iron deficiency in inflammatory bowel disease (IBD) remains a diagnostic challenge in a clinical routine. The aim of the study was to detect the most efficient marker of iron deficiency in IBD children. In a group of 75 IBD children, we evaluated the sensitivity, specificity, accuracy, and positive and negative predictive values of erythrocytes’ indices, including MCV, MCH, MCHC and RDW, and biochemical markers, including iron, transferrin, sTfR and sTfR/log ferritin, for identifying iron deficiency. Receiver operating characteristic (ROC) analysis was used to compare the ability of these parameters to detect iron deficiency. The best predictors of iron deficiency were sTfR/log ferritin, with accuracy 0.86, sensitivity 0.98, specificity 0.63, positive predictive value 0.83 and negative predictive value 0.94, and sTfR, with accuracy 0.77, sensitivity 0.82, specificity 0.67, positive predictive value 0.82 and negative predictive value 0.67. Moreover, sTfR/log ferritin exhibited the largest area under ROC (0.922), followed by sTfR (0.755) and MCH (0.720). The sTfR/log ferritin index appears to be the most efficient marker of iron depletion in pediatric IBD, and it may give an added value in the management of IBD patients.

## 1. Introduction

Iron deficiency is a widespread complication of inflammatory bowel disease (IBD) which may affect 36% to 90% of IBD patients, depending on the studied population and applied criteria of iron deficiency [1]. It has been found that, at IBD onset, about 90% of children with Crohn’s disease and 95% of children with ulcerative colitis suffered from depleted iron stores. Moreover, a two-year follow-up analysis showed that iron deficiency was still present in 70% of children with Crohn’s disease and 65% of children with ulcerative colitis [2]. Thus, it appears that, in children with IBD, iron deficiency may not be identified properly or not treated sufficiently.

There are several possible causes of iron depletion among IBD patients, including loss of blood from the gastrointestinal tract, limited iron supply and impaired iron absorption [3,4]. It has to be highlighted that increased hepcidin level in IBD is responsible for iron retention in enterocytes and macrophages that leads to inhibition of iron turnover, hypoferremia and iron-restricted erythropoiesis [3,5].

Deficiency of iron may be manifested as a variety of symptoms including impaired growth and cognitive function, malaise, headache, dizziness, dyspnea, alopecia, stomatitis, glossitis, restless leg syndrome and pica [3]. Moreover, disturbances in iron homeostasis may influence the immune system, affecting lymphocyte development and function [3,4]. Iron deficiency is also associated with thrombocytosis, which may increase the risk of thromboembolism [3]. Last but not least, progressing depletion of iron stores attenuates erythropoietic activity, which eventually leads to iron deficiency anemia and its complications [3,4]. Moreover, negative iron balance may exacerbate the course of IBD, leading to accelerated clinical deterioration [6].

Detection of the depletion of iron stores without anemia is a diagnostic challenge in chronic inflammatory diseases. Unspecific clinical manifestations of empty iron stores may mimic symptoms of IBD, which results frequently in overlooking iron deficiency in these patients. However, prompt recognition and appropriate management of iron deficiency is crucial because of the essential role of iron in a wide variety of metabolic processes [7]. Thus, it is advocated to perform routine screening tests for anemia and iron deficiency at least every three months in patients with active IBD, and every six to twelve months in IBD remission or in mild cases of the disease [8].

Decreased serum ferritin concentration is an early marker of iron depletion, and serum ferritin is widely used in the diagnosis of iron deficiency in generally healthy children [9]. However, since ferritin is also an acute phase reactant, it may be affected by the inflammation underlying IBD [10]. Thus, in the presence of inflammation, it is recommended to use serum ferritin and saturation of transferrin to estimate body iron stores [8]. Both parameters have certain limitations, including analytical and biological variability [7,10]. Several parameters have been suggested as reliable iron markers in inflammatory conditions. Among novel biomarkers, soluble transferrin receptor (sTfR) has been included. Soluble transferrin receptor (sTfR) is presented in circulation as a truncated form of transferrin transmembrane receptor [11]. The value of sTfR remains in proportional relationship to the density of TfR which is expressed on almost all body cells [10,11]. Thus, sTfR indicates the amount of iron which is available for erythropoiesis and constitutes a biomarker of functional iron compartment in the body [10]. Expression of transferrin receptors is determined by iron homeostasis, erythropoietic activity and hypoxia. In iron depletion and iron deficiency anemia there is an increase of sTfR. On the other hand, hyperferremia leads to a decrease of sTfR [11]. In conditions with hypoploriferative erythropoiesis, including aplastic anemia or chronic kidney disease, the number of sTfR is diminished, while enhanced erythropoiesis in the course of hemolytic anemia is associated with increased sTfR [11]. Last but not least, hypoxia may augment the level of sTfR in the circulation [11]. However, the expression of sTfR is not affected by inflammation, which determines its superiority over classical markers of iron homeostasis used in clinical practice in patients with chronic inflammatory diseases [10]. Thus, sTfR may serve to distinguish iron deficiency anemia from anemia of chronic diseases in these patients [10,11]. Moreover, its index sTfR/log ferritin combines two dimensions of iron homeostasis, i.e., its functional and storage pool, and is assumed to assess even more precisely the character of anemia in chronic inflammatory diseases than solely sTfR [10,12].

Nevertheless, to the best of our knowledge, there appears to exist no study assessing the diagnostic performance of traditional and alternative parameters of iron status in children with IBD. The aim of this study was to compare the diagnostic utility of erythrocytes’ indices, including MCV, MCH, MCHC, RDW, and biochemical markers, including iron, transferrin, sTfR, sTfR/log ferritin, in the recognition of iron depletion in IBD children.

## 2. Materials and Methods 

In the study group, there were 75 consecutive patients with IBD hospitalized at the Department of Pediatrics and Gastroenterology (formerly named the Department of Pediatrics), Medical University of Lublin, Poland from February 2013 to August 2015. Exclusion criteria were: taking iron supplements and/or an erythropoiesis-stimulating agent within the previous 3 months; blood transfusion within 4 weeks preceding the enrolment; acute infection at the time of recruitment and/or within 4 previous weeks; any concomitant chronic disease.

In all patients, we collected blood samples for a complete blood count, serum iron, ferritin, transferrin, saturation of transferrin (satTf) and soluble transferrin receptor (sTfR).

The sTfR/log ferritin index was calculated by the following formula [12]:

(1) sTfRlogferritin=sTfR [mgL]log10ferritin [ngmL].

Iron deficiency was defined as a decrease of serum ferritin below 30 ng/mL in IBD remission, and as satTf below 20% with ferritin below 100 ng/mL in the active phase of IBD [3,8]. 

For statistical analysis, we used Statistica v. 12.0 (StatSoft, Poland). To compare variables between the two groups, a Mann-Whitney U-rank test was used. Differences were considered statistically significant for a *p*-value if < 0.05. Measures of diagnostic accuracy, including sensitivity (SENS), specificity (SPEC), accuracy (ACC), positive predictive value (PPV) and negative predictive value (NPV), were calculated for erythrocytes’ indices (MCV, MCH, MCHC, RDW) and biochemical parameters (iron, transferrin, sTfR, sTfR/log ferritin) for predicting iron depletion in IBD patients. Receiver operating characteristic (ROC) analysis was used to establish the diagnostic utility of tested parameters to detect iron deficiency. Area under the receiver operating curve (AUROC) with 95% confidence intervals was determined. Cut-points values were chosen to maximize the sensitivity and specificity of analyzed parameters.

All subjects’ parents and patients aged ≥16 years old provided written informed consent for participation in this study. Ethical approval for this study was obtained from the Bioethics Committee of Medical University of Lublin (KE–0254/22/2013).

The study was funded by the Medical University of Lublin; Grant number MNsd466.

## 3. Results

The study group consisted of 75 IBD patients, including 46 (61.3%) children with ulcerative colitis and 29 (38.7%) children with Crohn’s disease. In the active phase of IBD there were 54 (72%) patients, while 21 (28%) were in IBD remission. There was a slight male predominance (*n* = 40; 53%). The mean age was 12.8 ± 3.7 years old (median: 13.5 years old; range: 3.5–18 years old). A detailed description of the study group was published previously [13,14].

Based on the applied criteria, iron deficiency was diagnosed in 50 (66.7%) subjects. Among these children, 33 (44%) fulfilled World Health Organization criteria for anemia, while 17 (22.7%) had iron deficiency without anemia. The frequency of iron deficiency did not differ significantly between patients with ulcerative colitis and Crohn’s disease (*χ*^2^ = 1.38; *p* = 0.24). We identified iron depletion in 33 out of 46 (71.7%) children with ulcerative colitis and 17 out of 29 (58.6%) children with Crohn’s disease. Moreover, iron deficiency was recognized in 70.4% of patients with active phase IBD and 57.1% of patients in IBD remission. However, that difference was not statistically significant (*χ*^2^ = 1.19; *p* = 0.27). Table 1 presents a comparison of tested parameters among children with iron deficiency and with normal iron supply. 

**Figure 1:**
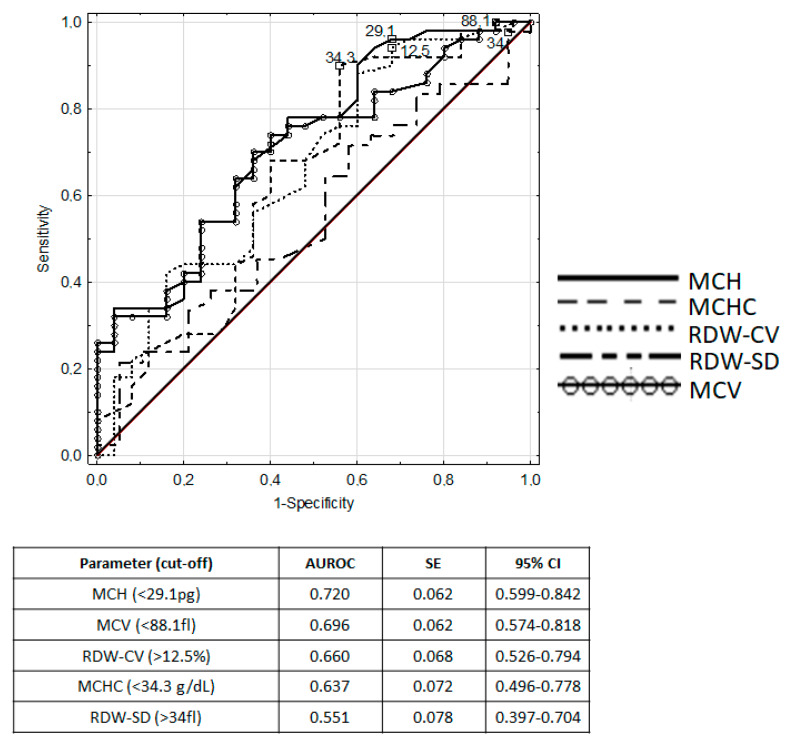
ROC curves and area under ROC for red blood cell indices in iron deficiency recognition in IBD children.

For iron deficiency detection, receiver operating characteristic (ROC) analysis was carried out. The ROC curves of red blood cell indices (MCV, MCH, MCHC, RDW) are plotted in Figure 1. 

**Figure 2:**
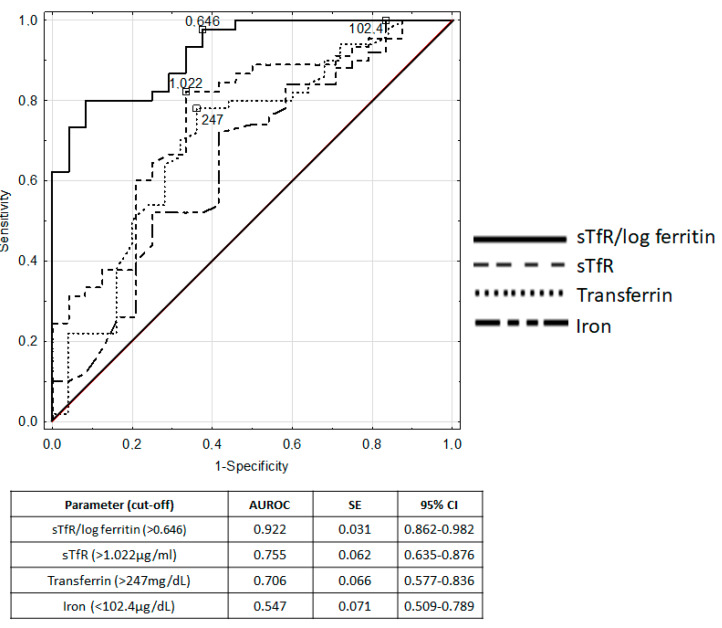
ROC curves and area under ROC for biochemical markers of iron deficiency recognition in IBD children.

Figure 2 presents ROC curves for biochemical markers of iron homeostasis, including serum iron, transferrin, sTfR and sTfR/log ferritin. 

The sTfR/log ferritin index had an excellent diagnostic utility to detect iron depletion in children with IBD, with an area under ROC (AUROC) of 0.922. Good diagnostic performance was characteristic for sTfR (AUROC 0.755), MCH (AUROC 0.720) and transferrin (AUROC 0.706). Table 2 presents the measures of diagnostic accuracy of analyzed parameters. 

Moreover, while determining iron depletion in patients with active IBD, sTfR/log ferritin also exhibited the greatest area under ROC (AUROC 0.908; SE 0.041; 95% CI 0.827–0.989), with a cut-off value 0.965. Its measures of diagnostic accuracy were as follows: sensitivity 0.76, specificity 0.93, accuracy 0.82, PPV 0.96 and NPV 0.64. Patients in IBD remission also showed good diagnostic utility in discriminating iron deficiency. Using a cutoff value of 1.068 for sTfR/log, ferritin was characterized with AUROC 0.939 (SE 0.052; 96%CI 0.838–1.0), sensitivity 0.91, specificity 0.89, accuracy 0.90, PPV 0.91 and NPV 0.89.

## 4. Discussion

The problem of iron deficiency is a significant issue in the population of children with IBD. Revel-Vilk et al. recognized iron deficiency in 42% of children and adolescents with IBD [15]. We found that iron deficiency is slightly more common in patients with ulcerative colitis than in Crohn’s disease; however, it was not statistically significant. Our results are in line with previous studies [2,16]. Moreover, iron deficiency was not related to disease activity. Wiskin et al. also found that iron deficiency occurred with almost the same frequency in children with newly diagnosed ulcerative colitis and Crohn’s disease (95% and 90% respectively) [2]. Among Brazilian outpatients with IBD, iron deficiency was present in 53.5% of patients with ulcerative colitis and 45.4% of patients with Crohn’s disease [16]. 

The most accurate measure of iron deficiency appears to be a statement of diminished or absent stainable iron in bone marrow aspirate [17,18]. However, this procedure is invasive, expensive and may require sedation, which attenuates its widespread utility in a clinical routine practice. Moreover, it has also limited usefulness in pediatric patients because the iron is not stored as marrow hemosiderin in infants and small children [17]. Thus, indirect peripheral biomarkers are used to evaluate iron homeostasis [10,17]. Decreased serum ferritin is a good indicator of pre-latent iron deficiency while iron stores are depleted. Serum iron, transferrin and saturation of transferrin correspond well to circulating iron and may be used to recognize latent iron deficiency with diminished erythropoiesis [17]. However, all of the above mentioned iron indices are affected not only by disturbances of iron metabolism, but also by acute and chronic inflammatory processes [10]. That fact provide an explanation to why routinely used iron parameters are not reliable in patients with inflammatory bowel diseases, and justify the need for research on biomarkers which are accurate and not dependent on inflammation.

Therefore, our objective was to demonstrate a comparison of seven conventional hematological and biochemical iron markers, and two novel parameters of iron homeostasis, i.e., sTfR and sTfR/log ferritin, in the population of children with IBD. We presented that sTfR/log ferritin >0.646 indicated iron deficiency with 98% sensitivity and 63% specificity. Moreover, ROC analysis showed the superior diagnostic performance of sTfR/log ferritin over other examined parameters in the detection of iron depletion in a population of IBD children. The sTfR, MCH and transferrin indicated moderate accuracy in discriminating children with IBD with iron deficiency from iron-replete children with IBD. Other classical parameters had poor diagnostic performance in detecting iron deficiency in pediatric IBD. Our results imply that sTfR/log ferritin may be the most efficient biomarker to diagnose iron deficiency among pediatric patients with IBD. Furthermore, a recent study by Abitbol et al. concluded that the use of sTfR/log ferritin in addition to serum ferritin increased diagnosis rates of iron deficiency by 36% in adults with IBD [19]. The sTfR/log ferritin index appears to be a complete biomarker of iron status in the human body, reflecting both the functional and storage iron pools [20]. While sTfR measurement indicates the degree of iron availability for cells, serum ferritin concentration is an indirect estimate of iron stores [10]. Previously, we have shown that sTfR/log ferritin was not dependent on serum inflammatory indices and the clinical activity of IBD [14]. Furthermore, sTfR/log ferritin demonstrated a better diagnostic efficacy than solely sTfR in the detection of iron deficiency anemia in pediatric patients with IBD [14]. These results were in accordance with previous findings reported in adults with IBD by Oustamanolakis et al. [21].

In different populations, it has been reported that sTfR/log ferritin is more useful than solely sTfR to recognize iron deficiency [22,23,24]. Previously, it has been shown that sTfR/log ferritin may be an early indicator of latent iron deficiency in adults [24]. Moreover, it was evidenced that, in a group of healthy children, sTfR/log ferritin exhibited a good diagnostic accuracy in detecting iron deficiency in the absence of anemia. The sensitivity of sTfR/log ferritin for iron depletion diagnosis using a cut-off of 2 was 89%, while specificity was 96%, positive predictive value was 90% and negative predictive value was 94% [22]. Furthermore, among healthy Malawian children, it has been found that sTfR/log ferritin was the most accurate parameter discriminating children with iron depletion from those with normal iron stores. Using the cut-off of sTfR/log ferritin >1.85, the sensitivity was 70.3%, specificity was 75%, positive predictive value 68.4% and negative predictive value was 76.6% [25].

In a group of 442 adults with various disease-specific anemias, sTfR/log ferritin was found to be the most accurate indicator of functional iron deficiency. It should be highlighted that sTfR/log ferritin had greater sensitivity and specificity in patients solely with iron depletion, compared to those with combined iron depletion and acute-phase response. Moreover, the sTfR/log ferritin cut-offs for iron deficiency differed depending on CRP concentration. In patients with CRP < 5 mg/L, sTfR/log ferritin cut-off was 1.5, while in those with CRP > 5 mg/L that value was 0.8 [23]. The possible explanation of the lower cut-off value for sTfR/log ferritin in patients with iron depletion accompanied by acute phase response was hypothesized to be that functional iron deficiency in the presence of hypoproliferative erythropoiesis does not cause an adequate elevation in sTfR compared with the extent of iron deficiency [23]. 

The index sTfR/log ferritin also appears to be a good indicator of iron deficiency in other diseases. It has been shown that, for distinguishing iron-deficient from iron-replete patients with rheumatoid arthritis, sTfR/log ferritin has an area under ROC of 0.97, sensitivity of 100% and specificity of 94% [26]. It has been also presented that sTfR/log ferritin index is useful in the identification of functional iron deficiency in adults with rheumatoid arthritis [27]. Moreover, sTfR/log ferritin was superior to conventional tests for predicting the response to intravenous iron supplementation in hemodialysis adults [28]. On the other hand, interpretation of sTfR and its indices in patients with chronic kidney disease should be cautious, and one should take into consideration the fact that sTfR revealed a relationship with glomerular filtration rate in patients in the early stages of chronic kidney disease and absolute iron deficiency [29].

Although sTfR/log ferritin appears to be a valuable biomarker of iron deficiency, the main limitation of its use in clinical practice is the lack of standardization of sTfR assays, and thus the lack of established reference ranges [30].

Our study has several limitations, including the relatively small study group and the homogenic population of children from a single pediatric center. Another concern about our findings is that the reference tests (ferritin and satTf) for iron deficiency were indirect. However, we assumed that bone marrow aspiration is not rational for the assessment of iron status in children with IBD because of the invasive character of that procedure.

## 5. Conclusions

To conclude, our results revealed that sTfR/log ferritin index appears to be the most efficient biomarker of iron depletion in IBD children. Conventional parameters of iron homeostasis are of limited value in iron deficiency recognition in that group of patients. The use of sTfR/log ferritin may give an added value to the diagnostics of iron deficiency in children with IBD and those with other chronic inflammatory diseases. However, further studies are needed to confirm our results and determine reliable reference ranges of sTfR/log ferritin before attempting to put the findings into practice.

## Figures and Tables

**Table 1:** Comparison of hematological and biochemical parameters among IBD patients with iron deficiency and normal iron supply.

Parameter	IBD Patients with Deficiency of Iron	IBD Patients without Iron Deficiency	*p*
**Hb (g/dL)**	11.51 ± 1.66	12.79 ± 1.59	*p* = 0.0009
**Ht (%)**	34.88 ± 4.41	38.16 ± 3.79	*p* = 0.002
**RBC (×10^6^/μL)**	4.632 ± 0.55	4.695 ± 0.50	*p* = 0.84
**MCV (fL)**	76.24 ± 6.52	81.23 ± 6.06	*p* = 0.006
**MCH (pg)**	24.93 ± 2.89	27.33 ± 2.76	*p* = 0.002
**MCHC (g/dL)**	32.63 ± 1.49	33.46 ± 1.66	*p* = 0.05
**RDW-SD (fl)**	40.38 ± 4.85	39.41 ± 4.27	*p* = 0.53
**RDW-CV (%)**	14.95 ± 1.83	13.95 ± 2.06	*p* = 0.02
**Iron (μg/dL)**	34.12 ± 26.04	53.03 ± 39.33	*p* = 0.04
**Transferrin (mg/dL)**	277.24 ± 54.87	234.44 ± 60.20	*p* = 0.003
**SatTf (%)**	8.42 ± 5.98	15.36 ± 10.10	*p* = 0.0006
**Ferritin (ng/mL)**	14.02 ± 13.260	73.80 ± 44.37	*p* < 0.0001
**sTfR (μg/mL)**	1.67 ± 0.99	1.06 ± 0.36	*p* = 0.0005
**sTfR/log ferritin**	2.50 ± 2.93	0.61 ± 0.21	*p* < 0.0001

**Table 2:** Measures of diagnostic utility of analyzed parameters for discriminating iron deficiency in children with IBD.

Parameter [cut-off]	Sensitivity	Specificity	Accuracy	Positive Predictive Value *p*	Negative Predictive Value
**MCV (<88.1 fL)**	1.00	0.08	0.69	0.69	1.00
**MCH (<29.1 pg)**	0.96	0.32	0.75	0.74	0.80
**MCHC (<34.3 g/dL)**	0.90	0.44	0.75	0.76	0.69
**RDW-SD (>34 fL)**	0.98	0.05	0.69	0.70	0.50
**RDW-CV (>12.5%)**	0.94	0.32	0.73	0.73	0.73
**Iron (<102.4 μg/dL)**	1.00	0.17	0.73	0.71	1.00
**Transferrin (>247 mg/dL)**	0.78	0.64	0.73	0.81	0.59
**sTfR (>1.022 μg/mL)**	0.82	0.67	0.77	0.82	0.67
**sTfR/log ferritin (>0.646)**	0.98	0.63	0.86	0.83	0.94
